# Stem cell derived basal forebrain cholinergic neurons from Alzheimer’s disease patients are more susceptible to cell death

**DOI:** 10.1186/1750-1326-9-3

**Published:** 2014-01-08

**Authors:** Lishu Duan, Bula J Bhattacharyya, Abdelhak Belmadani, Liuliu Pan, Richard J Miller, John A Kessler

**Affiliations:** 1 Departments of Neurology, Northwestern University’s Feinberg School of Medicine, Feinberg School of Medicine, 303 East Chicago Avenue, Chicago, IL 60611-3008, USA; 2 Molecular Pharmacology and Biological Chemistry, University’s Feinberg School of Medicine, 303 East Chicago Avenue, Chicago, IL 60611-3008, USA

**Keywords:** Alzheimer’s disease, Human induced pluripotent stem cells, Basal forebrain cholinergic neurons, Aβ42/40 ratio, Aβ rise, Glutamate excitotoxicity, Calcium abnormalities

## Abstract

An early substantial loss of basal forebrain cholinergic neurons (BFCNs) is a constant feature of Alzheimer’s disease (AD) and is associated with deficits in spatial learning and memory. Induced pluripotent stem cells (iPSCs) derived from patients with AD as well as from normal controls could be efficiently differentiated into neurons with characteristics of BFCNs. We used BFCNs derived from iPSCs to model sporadic AD with a focus on patients with *ApoE3/E4* genotypes (AD-E3/E4). BFCNs derived from AD-E3/E4 patients showed typical AD biochemical features evidenced by increased Aβ42/Aβ40 ratios. AD-E3/E4 neurons also exhibited altered responses to treatment with γ-secretase inhibitors compared to control BFCNs or neurons derived from patients with familial AD. BFCNs from patients with AD-E3/E4 also exhibited increased vulnerability to glutamate-mediated cell death which correlated with increased intracellular free calcium upon glutamate exposure. The ability to generate BFCNs with an AD phenotype is a significant step both for understanding disease mechanisms and for facilitating screening for agents that promote synaptic integrity and neuronal survival.

## Background

Alzheimer’s disease (AD) is a progressive debilitating neurodegenerative disorder that typically occurs in the elderly. The majority of AD cases are sporadic without any known genetic mutations. However polymorphism in the *Apolipoprotein E* (*ApoE*) gene is a strong risk factor for AD. Compared to individuals with an *ApoE3/E3* genotype, the presence of one copy of the *E4* allele increases AD risk by 2 to 3 fold, and two copies of *E4* increases risk up to 12 fold [1,2].

The etiology of AD is poorly understood, but there are consistent pathologic features of diseased brains including senile plaques composed of β-amyloid [3,4], and neurofibrillary tangles formed by hyperphosphorylated tau [5]. β-amyloid plaques are comprised of aggregated, extracellularly deposited Aβ peptides. Aβ peptides are typically 39–42 amino-acids long and are generated from amyloid precursor protein (APP) by sequential β- and γ-secretase cleavages. Aβ40 is normally the major form of secreted Aβ peptide recovered from cerebrospinal fluid, while Aβ42 represents less than 10% [6]. However, in AD, the more amyloidogenic Aβ42 is significantly elevated and is hypothesized to be the initial and predominant species found in plaques [7]. The progressive cognitive decline of AD is a consequence of loss of synapses and eventually neurons in basal forebrain, cortex and hippocampus [8,9]. Basal forebrain cholinergic neurons (BFCNs) are the predominant source of cortical cholinergic input and play a central role in spatial learning and memory. AD-related tauopathies arise earliest in cholinergic neurons of the basal forebrain and loss of these neurons parallels cognitive decline [10,11]. For these reasons this population of neurons is an ideal target for the study of the cellular pathophysiology of AD.

Study of Alzheimer’s disease has been limited in the past by the lack of availability of live neurons derived from AD patients. However induced pluripotent stem cells (iPSCs) can be derived from human skin fibroblasts or other easily accessible tissues and can then be differentiated into neurons [12,13]. Mixed neuronal cultures derived in such a way from AD patients displayed some biochemical features of the disease including increased Aβ42/40 ratios, elevated levels of Aβ42 or Aβ40, and increased phosphorylation of tau [14-16]. However the abnormalities in these studies were largely demonstrated for familial AD caused by genetic mutations in *presenilin* or *APP*, and disease manifestations were highly variable in the limited sporadic AD lines that were examined.

We previously developed a protocol to generate a high percentage of BFCNs from human embryonic stem cells (hESCs) [17]. In this report we find that the same techniques can be used to generate BFCNs with high efficiency from iPSCs. We focused on sporadic AD with an *ApoE3/E4* genotype and found that BFCNs derived from such patients display biochemical abnormalities associated with the disease and are more susceptible to both glutamate- and calcium- mediated cell death.

## Results

### Generation of iPSCs from human control and Alzheimer’s disease fibroblasts

Age matched human fibroblasts were purchased from Coriell institute from either healthy controls or Alzheimer’s disease patients with *ApoE3/E4* genotypes. iPSCs were generated with a polycistronic retroviral vector encoding Klf4, Oct4, Sox2 and c-Myc (Additional file 1: Figure S1). Individual colonies were picked and expanded as separate lines. We established control iPSCs lines from the following subjects: control1, a 43-year-old female; control2, a 71-year old female; control3, a 61-year old male; an iPSCs line from WiCell (iPS-DF6-9-9T) was used as a fourth control. Sporadic Alzheimer’s disease iPSC lines with *ApoE3/E4* genotypes included: AG05810, a 79-year old female with late AD onset; AG04402, a 47-year old male with early AD onset; and AG11414, a 39-year old male with early AD onset. We also included two familial AD lines in some of our studies as comparators: AG06848, a 56-year-old female with a *presenilin1* point mutation and AG07872, a 53-year-old male AD patient with genetic mutations. A complete list of iPSCs lines we used is provided in Table 1.

**Table 1:** List of iPSCs

**Name**	**Gender**	**Age taken**	**Figures presented**	**Vendor**	**Specification**
Control1	Male	61	1,2,3,4,5,S4,S5,S6	Coreill	Healthy control
Control2	Female	43	4,5,6,S4	ATCC	Healthy control
Control3	Female	71	4,5,S4	Coreill	Healthy control
Control4	Male	N/A	4	WiCell	Healthy control
AG04402	Male	47	4,5,6,S2,S4,S5	Coriell	AD, Early APOE3/E4
AG11414	Male	39	3,4,5,S2,S4,S5,S6	Coriell	AD, Early APOE3/E4
AG05810	Female	79	4,5,6,S4	Coriell	AD, Late APOE3/E4
AG07872	Male	53	4	Coriell	AD, Familial
AG066848	Female	56	4	Coriell	AD, PS1 (A246E)

All control and AD iPSCs lines showed typical human embryonic stem cell (hESC) morphology and maintained normal karyotypes during culturing (data not shown). Undifferentiated iPSCs all immunostained for the pluripotent stem cell markers Oct4, Sox2, SSEA4, andTra1-60 (Additional file 2: Figure S2A). When differentiated *in vitro* using embryoid body formation, both control and AD iPSCs gave rise to cell types of all three germ layers, as demonstrated by marker staining, Collagen type IV (mesoderm), Gata4 (endoderm) and Map2 (ectoderm) (Additional file 2: Figure S2B). Some lines were also tested for their ability to form teretaomas, and the tested lines were able to generate teratomas with features characteristic of mesoderm, endoderm and ectoderm (Additional file 2: Figure S2C), further confirming their pluripotent stem cell identity.

### Electrophysiologically active BFCNs generation from control and AD iPSCs

**Figure 1:**
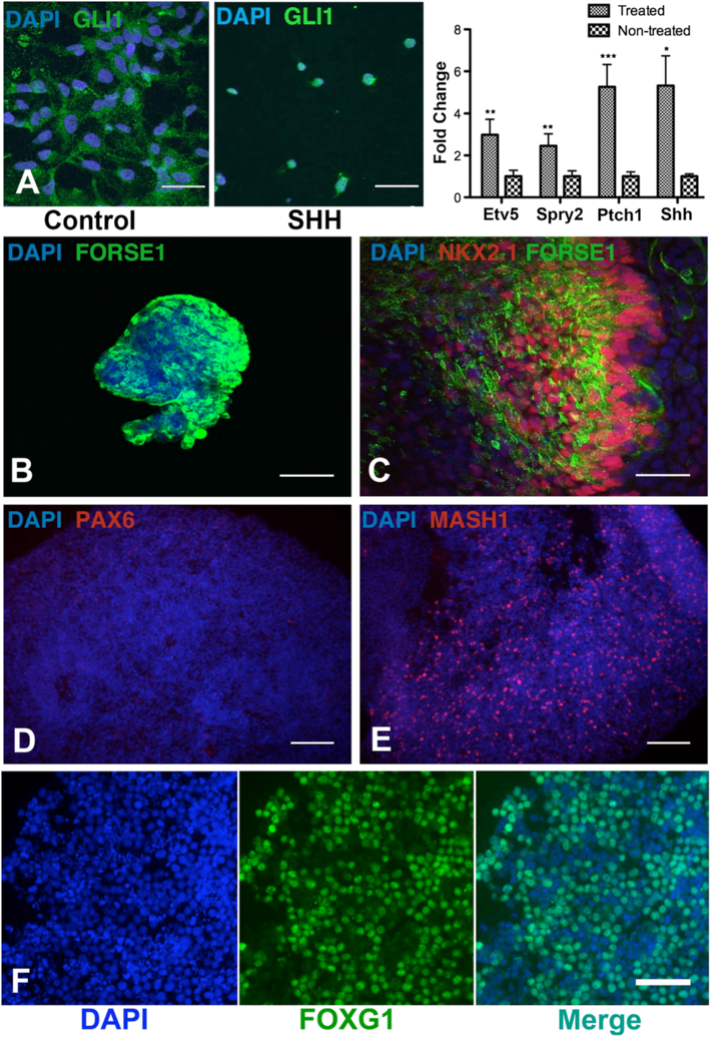
**Differentiation of iPSCs into ventral forebrain neural progenitors. (A)** 72 hours of SHH treatment of iPSCs (Control 1 line)-derived neural progenitors result in translocation of downstream effector Gli1 into the nucleus, Scale bar = 42 μm. Quantitative PCR shows up-regulation of the SHH downstream genes *Ptch1* and *Shh* and the FGF8 downstream genes *Etv5* and *Spry2* after 72 hours of SHH and FGF8 treatment of neurospheres. Data are normalized to non-treated controls (n = 4, mean ± SEM, two-way ANOVA, p = 0.0003, F(1,32) = 17.73; Bonferroni multiple comparisons, *p < 0.05, **p < 0.01, ***p < 0.001, n indicates number of replicative experiments). Immunocytochemistry analysis of SHH and FGF8 treated neurospheres for forebrain marker **(B)** Forse1, ventral markers **(C)** Nkx2.1 and **(E)** Mash1 and dorsal marker **(D)** Pax6. Scale bars = 85 μm. Nuclei are stained with DAPI in blue. **(F)** Immunocytochemistry of SHH and FGF8 treated neurospheres shows expression of the telencephalic marker FoxG1 which co-localizes in the nucleus with DAPI.

Our laboratory previously reported generation from hESCs of highly enriched cultures of cells with the characteristics of BFCNs [17]. Although iPSCs are similar to hESCs, they are not identical and can have differences such as differential promoter methylation states and gene expression patterns [18]. We therefore asked if the protocol used to generate BFCNs from hESCs could be applied to iPSCs. We treated iPSCs grown on feeder free matrigel matrix with retinoic acid for 7 days and dissociated cells for neurosphere formation. Robust neurosphere formation was seen with both control and AD iPSCs. Sonic hedgehog (SHH) and FGF8 signaling are involved in patterning BFCNs precursors in the medial ganglion eminence (MGE) during the development of ventral forebrain [19,20] and are required for BFCNs generation from hESCs [17]. We therefore asked if iPSCs responded to SHH and FGF8 signaling and if they differentiated accordingly into a ventral forebrain precursor phenotype. Treatment of control iPSCs-derived neurospheres with 200 ng/ml SHH and 100 ng/ml FGF8 for 72 hours resulted in translocation of the SHH downstream effector Gli1 into the nucleus (Figure 1A). Quantitative PCR demonstrated up-regulation of levels of the SHH downstream molecules *Ptch1* and *Shh*[21] and the FGF8 downstream molecules *Etv5* and *Spry2*[22,23] (Figure 1A). Thus iPSCs respond to SHH and FGF8 treatment by activating their appropriate downstream targets. To examine if the SHH/FGF8 treated iPSCs neurospheres were relevant progenitors for BFCNs, we immunostained the neurospheres for known MGE markers. SHH/FGF8 treated control iPSCs neurospheres expressed the forebrain marker Forse1 (Figure 1B) and the ventral markers Nkx2.1 and Mash1 (Figure 1C, E), but were negative for the dorsal marker Pax6 (Figure 1D). The cells also expressed the telencephalic marker FoxG1 (Figure 1F). Taken together, these results suggest that patterning iPSCs-derived neurospheres with SHH and FGF8 directed the cells towards a ventral forebrain progenitor phenotype, making them relevant progenitors for BFCNs.

**Figure 2:**
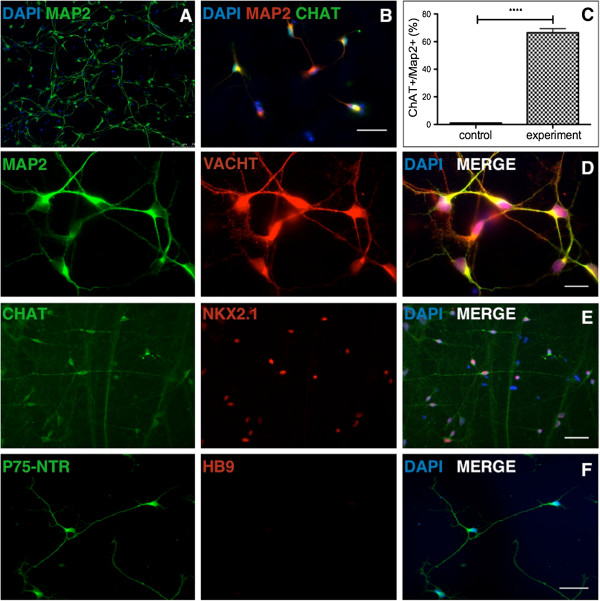
**Generation of basal forebrain cholinergic neurons from iPSCs. (A)** Over 90% of cells derived from SHH/FGF8 treated neural progenitors (Control 1 line shown) are Map2 positive neurons. **(B)** ChAT positive cholinergic neurons are generated after nucleofection of SHH/FGF8 treated neural progenitors with Lhx8/Gbx1 transcription factors. **(C)** Quantification of ChAT positive neurons among total Map2 positive neurons in SHH/FGF8 treated and Lhx8/Gbx1 nucleofected culture compared to non-treated, non-nucleofected control group (n = 3, Student’s t-test, ****p < 0.0001, n indicates number of replicative experiments). Further confirmation of basal forebrain cholingeric identity was done by staining with **(D)** VAChT **(E)** co-expression of ChAT with Nkx2.1 **(F)** expression of BFCN specific marker p75-NTR but not spinal cholinergic marker HB9. Scale bars = 16 μm for panel D and 85 μm for other panels. Nucleus is stained with DAPI in blue.

Two transcription factors, Lhx8 and Gbx1, are crucial for BFCNs specification from committed progenitors in mice [24,25], and we have previously demonstrated that Lhx8 and Gbx1 are both necessary and sufficient for BFCNs induction from hESC-derived neurospheres [17]. We therefore transiently expressed Lhx8/Gbx1-IRES-EGFP plasmid in dissociated SHH/FGF8 treated control iPSCs-derived neurospheres via nucleofection. 72 hours later, we used fluorescence-activated cell sorting (FACS) for EGFP to enrich for BFCNs progenitors which co-expressed Lhx8 and Gbx1 and then plated the cells for differentiation in neurobasal medium with nerve growth factor (NGF) (Additional file 3: Figure S3). Two weeks later, we examined the final subtype of neurons by immunostaining. More than 95% of cells were Map-2 positive neurons (Figure 2A). Approximately 66 ± 3% of these neurons expressed the cholinergic neuron marker choline acetyltransferase (ChAT) (Figure 2B, C) as well as another cholinergic marker, the vesicular acetylcholine transporter (VAChT) (Figure 2D). To further confirm the phenotype of these cells, we examined expression of two markers characteristic of BFCNs, the low affinity neurotrophin receptor p75 shown be to expressed by over 95% of BFCNs [26] and Nkx2.1 reported to persist in postmitotic BFCNs [27]. Immunostaining revealed co-localization of ChAT with Nkx2.1 (Figure 2E); and expression of p75, by contrast the cells did not express HB9, a cholinergic motor neuron marker (Figure 2F). There were no significant differences in the efficiency of differentiation of AD and control BFCNs either into ventral forebrain neural precursors or basal forebrain cholinergic neurons (Additional file 4: Figure S4). Thus we were able to differentiate both control and AD iPSCs into a BFCNs phenotype by nucleofection of specific transcription factors.

**Figure 3:**
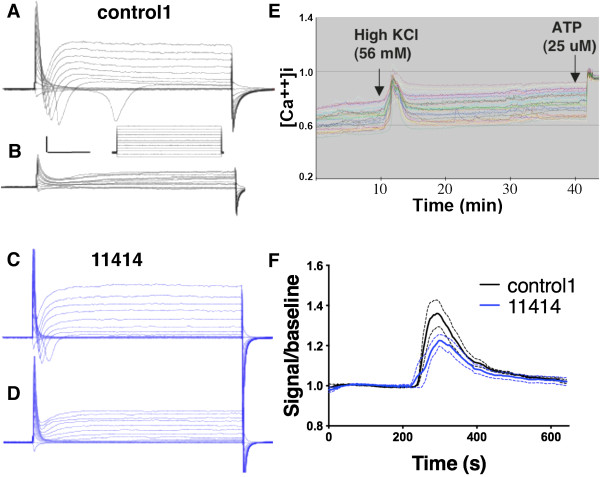
**Control and AD iPSCs-derived BFCNs express functional voltage-gated sodium and calcium channels.** Representative current traces evoked by step depolarization in the absence **(A, C)** or presence of TTX **(B, D)** from 2-week old control1 and *ApoE3/E4* AD line AG11414 BFCNs, note the elimination of the fast-inactivating inward current in the presence of TTX. Scale bar = 1 nA, 10 ms. **(E)** Calcium imaging with Fura-2 showed calcium transients evoked by depolarization with 56 mM KCl (black arrow indicates time of addition) of 2-week old BFCNs. ATP was added as a control for metabolically active neurons. **(F)** 2-week old control and AD BFCNs were briefly exposed to 56 mM KCl and calcium influx was monitored by calcium imaging. Peak amplitude from 25 cells were averaged and normalized to baseline for each group, dashed lines indicate SEM of each data point, n = 3, n indicates number of replicative experiments.

Voltage clamp recordings of 2-week old BFCNs cultures were performed to determine if the cells were electrophysiologically active in culture. Control neuron cultures showed voltage-activated currents (Figure 3A). The inward current was blocked by tetrodotoxin (TTX) (Figure 3B), a selective voltage-gated sodium channel blocker, demonstrating the presence of functional sodium channels. AD iPSCs-derived neurons similarly expressed tetrodotoxin-sensitive voltage-activated currents as illustrated by voltage clamp recordings in line AG11414 (Figure 3C, D). Depolarization of BFCNs with high KCl (56mM) elicited significant calcium influx in control neurons shown by calcium imaging with Fura-2 calcium dye (Figure 3E), indicating expression of voltage-gated calcium channels. AD neurons also expressed functional voltage-gated calcium channels and the amount of calcium influx through such channels upon KCl stimulation was not significantly different from control in the lines tested (Figure 3F, n = 3). The presence of voltage-gated calcium channels in both control and AD BFCNs were further confirmed by western blot using an L-type calcium channel antibody (Additional file 5: Figure S5). Taken together, these observations indicate that we were able to use our previously established protocol to differentiate both control and AD iPSCs into highly enriched cultures of neurons that expressed characteristic BFCNs markers and that were electrophysiologically active.

### AD-E3/E4 BFCNs have increased Aβ42/40 ratios and altered responses to γ-secretase inhibitors

**Figure 4:**
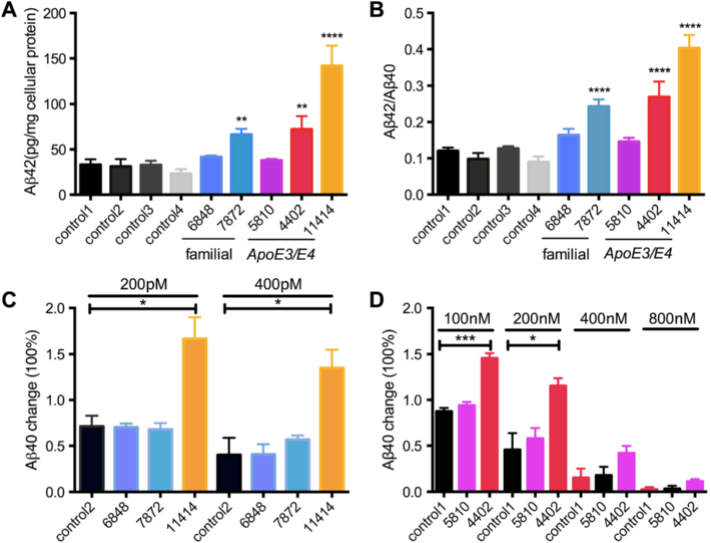
**AD-E3/E4 BFCNs have elevated Aβ42/Aβ40 ratios and altered responses to γ-secretase inhibitors.** Medium conditioned by 300,000 neurons for 72 hours was assayed simultaneously for soluble Aβ40 and Aβ42 by sandwich ELISA. Levels of Aβ secretion were normalized to cellular protein. **(A)** Aβ42 levels were significantly elevated in familial AD line AG07872 and *ApoE3/E4* lines AG04402 and AG11414 compared to the average of controls (n = 4, mean ± SEM, one-way ANOVA, p < 0.0001, F(5,36) = 24.16; Bonferroni multiple comparisons, **p < 0.01, ****p < 0.0001). **(B)** The ratios of secreted Aβ42 to Aβ40 were compared to the average of controls (n = 4, mean ± SEM, one-way ANOVA, p < 0.0001, F(5,36) = 34.70; Bonferroni multiple comparisons, ****p < 0.0001). **(C, D)** Quantification of Aβ40 secretion in response to different dosages of γ-secretase inhibitor compound E **(C)** or DAPT **(D)** treatment. (n = 3, mean ± SEM, Student’s t-test, *p < 0.5, ***p < 0.001), n indicates number of replicative experiments from the same line.

To test if our iPSCs-derived BFCNs from AD-E3/E4 patients display any disease related phenotypes, we examined Aβ secretion by the cells. In addition to the normal controls we also included two familial AD cases as potential positive controls. 300,000 neurons derived from each line were cultured on 12-well plates in 1 ml medium for 3 days and the medium was assayed simultaneously for Aβ42 and Aβ40 by sandwich ELISA. Cellular protein was extracted and measured by BCA protein assay to control for input cell number. ELISA results were read within the linear range of the standard curves and Aβ concentrations were calculated and normalized to cellular protein level. There were no significant differences in the amount of secreted Aβ40 among the lines, ranging between 200 and 300 picograms per milligram of cellular protein (data not shown). However Aβ42 secretion was significantly increased in several AD lines (Figure 4A). As a result, Aβ42/ Aβ40 ratios were significantly elevated in AD-E3/E4 lines AG04402 (~3 fold, n = 4, p < 0.0001) and AG11414 (~4 fold, n = 4, p < 0.0001) as well as in familial line AG07872 (~2.5 fold, n = 4, p < 0.0001) compared to the average of controls (Figure 4B). However, Aβ42/β40 ratios were not elevated in familial line AG06848 or in AD-E3/E4 line AG05810. We then examined the effects of treatment with the γ-secretase inhibitor, compound E (IC_50_ = 300 pM). At a concentration of 200 pM the γ-secretase inhibitor reduced Aβ40 production as expected in the familial and control lines that were tested (Figure 4C). Surprisingly, the treatment paradoxically increased Aβ40 secretion in the AD-E3/E4 line AG11414 by 67 ± 24% (Figure 4C). At a concentration of 400 pM the inhibitor further reduced production of Aβ40 in familial lines AG06848 (59 ± 11% reduction), AG07872 (43 ± 4% reduction), and control2 (60 ± 19% reduction), but it increased Aβ40 production from AG11414 (35 ± 20% increase). To determine whether this paradoxical response occurred in other sporadic AD lines and with other inhibitors, we tested another γ-secretase inhibitor DAPT (IC_50_ = 20 nM) in more lines. A similar shift in γ-secretase inhibitor potency in the AD-E3/E4 line AG04402 was observed. Aβ40 secretion was increased by 45 ± 6% and 15 ± 6% at 100 nM and 200 nM DAPT concentrations respectively in AG04402. By contrast, the inhibitor reduced secretion as expected in the control line and in AG05810. At higher concentrations, (400 nM and 800 nM) DAPT inhibited Aβ40 production from all lines including AG04402 (Figure 4D). Although individual variances were observed, these findings indicate that BFCNs derived from AD patients with an *ApoE3/E4* genotype demonstrate Aβ secretion abnormalities *in vitro.*

### AD-E3/E4 BFCNs show increased sensitivity to neurotoxic stimuli

**Figure 5:**
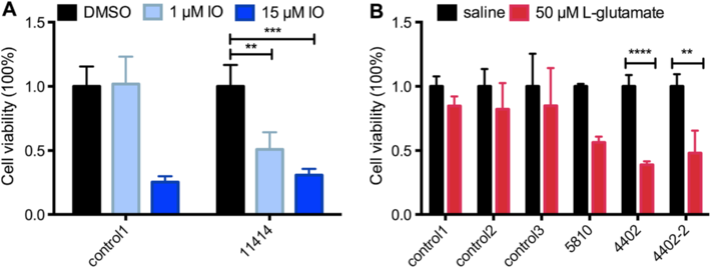
**AD-E3/E4 BFCNs are more sensitive to ionomycin or glutamate treatment. (A)** 2-week old BFCNs were treated with DMSO, 1 μM or 15 μM ionomycin for 16 hours, live/dead assay was performed and quantified, DMSO control is normalized to 1. (n = 3, mean ± SEM, two-way ANOVA, p = 0.0001, F(2,18) = 20.53; Bonferroni multiple comparisons, *p < 0.05, **p < 0.01, ***p < 0.001, ****p < 0.0001, n indicates number of replicative experiments from the same line). **(B)** 2-week old BFCNs were exposed to saline or 50 μM glutamate for 15 minutes and cells were incubated for another 24 hours. Cell viability was quantified by MTT assay. Both sets of data are presented as percentages of living cells. (n = 3, mean ± SEM, two-way ANOVA, p < 0.0001, F(1, 36) = 38.43; Bonferroni multiple comparisons, *p < 0.05, **p < 0.01, ***p < 0.001, ****p < 0.0001, n indicates number of replicative experiments from the same line).

BFCNs degenerate early in the course of AD. Since we have a system that allows us to directly assess human BFCNs survival, we compared AD-E3/E4 and control BFCNs for susceptibility to cell death. The precise mechanisms of neuronal death in AD are still unclear, but multiple proposed mechanisms ultimately converge on disrupted calcium signaling [28]. AD mutations have been reported to deregulate calcium signaling and sensitize neurons to apoptosis [29]. To determine whether AD-E3/E4 BFCNS are more susceptible to calcium-mediated cell death, 2-week old BFCNs were treated with three different doses of the calcium ionophore, ionomycin (250 nM, 1 μM or 15 μM) or with buffer (DSMO) for 16 hours, and a live/dead assay was performed to assess cell viability (Additional file 6: Figure S6). Neither the control nor AD groups had significant cell death after exposure to 250 nM ionomycin (data not shown). 1 μM ionomycin treatment had little effect on cell viability of control neurons, but it reduced cell viability by about 50% in AD-E3/E4 line AG11414 (Two-way ANOVA, n = 3, p < 0.01) (Figure 5A). High concentrations (15 μM) of ionomycin killed more than 70% of both control and AD neurons. Thus BFCNs derived from the AD-E3/E4 AG11414 died in response to low, normally non-lethal amounts of calcium influx.

Calcium ionophores make all membranes permeable to calcium, including plasma membranes, mitochondria and endoplasmic reticulum, which is unlikely to happen *in vivo*. To determine if sporadic AD-E3/E4 BFCNs are also more susceptible to more physiologically relevant neurotoxic stimuli, we examined their susceptibility to excitotoxic death in response to glutamate. 2-week old neuronal cultures were treated with either saline or 50 μM L-glutamic acid for 15 minutes, washed with PBS, and incubated with culture medium for additional 24 hours. The MTT assay, which takes advantage of color conversion of the MTT dye by mitochondrial succinate dehydrogenase of living cells, was used to quantify cell viability according to the manufacturer’s protocol. We included three controls and two iPSCs lines from the same sporadic AD patient AG04402 to address concerns about possible inter-line variance. The control BFCNs did not show a significant reduction in cell viability upon 50 μM glutamate exposure. Late-onset AD-E3/E4 line AG05810 had a 44% reduction is cell number. Cell survival after glutamate exposure was also significantly reduced in both lines independently derived from early onset AD-E3/E4 line AG04402; there was a 62% reduction in cell numbers in the line labeled as 4402 and a 53% decrease in line 4402–2 (Two-way ANOVA, n = 3, p < 0.0001 and p < 0.01 respectively) (Figure 5B). Thus BFCNs derived from AD-E3/E4 iPSCs displayed increased susceptibility to glutamate-induced excitotoxic death. The data with two different lines from the same AD-E3/E4 patient, combined with the consistency of phenotypes in multiple control lines, suggest that the observed difference is unlikely to be due to differences in the creation of the iPSCs, but rather reflects intrinsic genetic and disease stage associated abnormalities.

### AD-E3/E4 BFCNs show increased cytoplasmic calcium levels upon glutamate exposure

**Figure 6:**
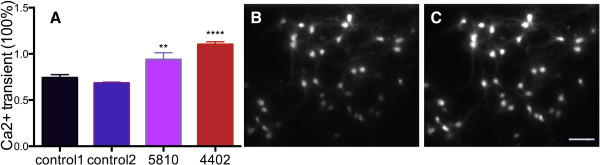
**AD-E3/E4 BFCNs have increased cytoplasmic calcium levels upon glutamate stimulation.** 2-week old neurons were briefly exposed to 50 μM glutamate and responses were recorded by calcium imaging and measured by ratio of signals at 340 nm/380 nm. Baseline and peak amplitude is measured for each individual cell and cytosolic free calcium levels were calculated as (peak amplitude-baseline)/baseline. **(A)** Averages of calcium transient of 20–30 cells per experiment were calculated and compared to the average of controls (n = 4, mean ± SEM, ANOVA, p = 0,0005, F(3, 12) = 19.55; Bonferroni multiple comparisons, **p < 0.01, ****p < 0.001), n indicates number of replicative experiments from the same line. Representative images of imaged cells at 340nm **(B)** and 380nm **(C)** are shown. Scale bar = 85 μm.

Neuronal excitotoxicity is thought to be mediated by calcium dependent processes [30]. To test if calcium signaling differences were associated with the differential susceptibility of control versus AD-E3/E4 BFCNs to glutamate, we examined calcium dynamics using Fura-2 calcium imaging. 2-week old neurons were incubated with 2 μM Fura-2 for 30 minutes, washed and incubated for another 30 minutes before imaging. Magnesium was excluded from all buffers to relieve the voltage-dependent block of NMDA receptors. Baseline level signals were established for 10 minutes before 50 μM glutamate was added briefly for 1 minute and the ratio of 340 nm to 380 nm was recorded. An enhanced calcium signal was seen strongly in both the cell bodies and in neuronal processes (Figure 6B and C). The calcium transient was quantified by subtracting the baseline from the peak value and then dividing by the baseline value for each individual neuron. 20–30 neurons from each experiment were analyzed and the responses were averaged. The control lines had approximately 70% increases of free intracellular calcium when briefly exposed to glutamate. By contrast, AG05810 neurons had an increase of over 90%, and AG04402 had about 110% increase in intracellular calcium levels (Figure 6A). The calcium transient was significantly higher in AD-E3/E4 lines AG05810 and AG04402 compared to the average of calcium transient in controls (One-way ANOVA, n = 3, p < 0.01 and p < 0.0001 respectively). We also measured resting free calcium concentrations and found no statistically significant differences among the lines examined. Control neurons had free cytosolic calcium concentrations ranging between 50-80 nM, whereas concentrations for AG05810 were between 90-120 nM, and for AG04402 between 108-162 nM. These observations suggest that the increased sensitivity of AD-E3/E4 neurons to glutamate toxicity may reflect elevated cytoplasmic calcium levels upon stimulation.

## Discussion

The ability to generate BFCNs with an AD phenotype is a significant step both for understanding disease mechanisms and for facilitating screening for agents that promote synaptic integrity and neuronal survival. Generation of relatively pure subpopulations of any type of neurons from iPSCs is difficult, and differentiation of BFCNs required a detailed protocol that was first established for the differentiation of hESCs [17]. Some approaches for the differentiation of hESCs and iPSCs are inefficient for generating BFCNs [31], and we used an approach that included retinoic acid followed by specific factors to induce a forebrain phenotype (SHH and FGF8) and nuclefection with two transcription factors (Lhx8 and Gbx1) that specify the BFCNs phenotype. Although retinoic acid (RA) may have some caudalizing effects, its use for generating forebrain precursors is well established [32,33]. SHH and FGF8 pretreated neural progenitors immunostained for the ventral marker Nkx2.1 and forebrain specific marker Forse1 [34], confirming the presence of a ventral forebrain progenitor population. The cells also expressed the forebrain marker FoxG1 which is initially detected in the cytoplasm [17] followed by translocation to the nucleus (Figure 1) as the cells begin to differentiate. Finally the cells expressed all of the known markers of the BFCNs phenotype and were electrically excitable. Although the protocol involved directed differentiation of the iPSCs, it is likely that the very high final purity of the cultures of BFCNs partially reflected selection due to early AraC treatment to eliminate proliferating cells and later NGF treatment to promote survival of BFCNs. These cultures should be useful not only for studying disease mechanisms but also for drug screening. For example, the glutamate excitotoxicity assay with MTT reagent (Figure 5) provides a direct readout for neuronal death, so that scaling the cultures to a 96 or 384-well format would provide a method for high-throughput screening for drugs that promote BFCNs survival.

There have been at least three prior studies that utilized iPSCs to study AD, but all examined heterogeneous mixtures of differentiated cells [14-16]. None of those studies examined cells from AD-E3/E4 patients, but they did examine cells from a few cases of sporadic AD and found great inconsistency in the phenotypes *in vit*ro. Israel et al. reported increased Aβ40 secretion in one sporadic line (*ApoE3/E3*) but not the other [15]. Kondo et al. found that only one of the two sporadic cases (genotype not known) had neuronal Aβ oligomer accumulation [16]. Some of the variability in those studies may reflect the heterogeneous, uncharacterized phenotypes of the differentiated cells that were examined. However heterogeneity was also observed in our study that focused on BFCNs. Although the findings with control cultures were remarkably consistent and reproducible across the four control lines used in our study, there were differences among the AD-E3/E4 lines. The AG11414 (*ApoE3/E4*) and AG04402 (*ApoE3/E4*) lines both differed significantly from controls in terms elevation of Aβ42/Aβ40 ratios, a paradoxical response to γ-secretase inhibitors, and increased sensitivity to glutamate induced toxicity. However the AG05810 (*ApoE3/E4*) line did not differ significantly from the controls in these respects. The reasons for these disparate findings are not yet clear, but there are a number of possibilities. The ages of onset of disease for AG11414 (35 y.o.) and AG04402 (47 y.o. and possibly earlier) were substantially younger than for AG05810 (79 y.o.), so the difference might reflect differences between early onset versus late onset disease. It is also possible that the lines produce different amounts of ApoE4 protein. It is well recognized that the *ApoE4* allele accelerates the onset of AD [35], and the late onset for AG05810 suggests less of an effect of the *ApoE4* allele. We attempted to find out if there was differential ApoE4 protein expression compared to ApoE3 protein level among these lines by western blot using a commercially available ApoE4 specific antibody. Unfortunately the antibody was not specific and recognized ApoE3 protein as well. Inter-line variance could also account for some of the differences since we used retrovirus to generate our iPSCs, and genome integration could lead to clonal heterogeneity and potential functional diversity [36]. However we tested this by examining two different independently derived lines from the same patient and found no significant differences. Coupled with the findings by other investigators, our observations suggest that it is likely patient specific factors rather than differences in reprogramming among lines account for the observed phenotypes.

As a major risk factor in sporadic AD, the *ApoE4* allele is associated with earlier onset and increased amyloid plaques in AD [37-39]. Animal studies demonstrated an isoform-dependent (*ApoE4* > *ApoE3* > *ApoE2*) effect on Aβ deposition [40,41]. ApoE4 also induced neuronal Aβ42 accumulation in target replacement ApoE4 mice [42]. Since astrocytes are the primary sources of secreted ApoE in mice, and have been shown to bind and degrade amyloid deposits [43,44], the above effects are thought to be primarily mediated by astroctyes. However our cultures contained no detectable astrocytes, and almost all of the cells expressed neuronal traits. Thus the effects of the *ApoE4* allele in this study were not mediated by astrocytes. Neurons also express ApoE [45,46], and our data suggest that neuronal ApoE4 increases secretion of Aβ42, the more fibrillar form that is more likely to aggregate and act as the core for amyloid plaques. In addition, we found an interesting shift of γ-secretase inhibitor potency in *ApoE3/E4* AD neurons. Low concentrations of γ-secretase inhibitors significantly increased Aβ secretion from *ApoE3/E4* neurons, in contrast to the predicted reduction of Aβ seen in control and familial AD lines. Such an Aβ rise at sub-threshold concentrations of γ-secretase inhibitors has been described in cell culture [47,48] and *in vivo* in human [49]. The underlying biochemical mechanism is not fully understood, but it is thought to be a direct γ-secretase effect [50,51]. Our finding that *ApoE* also plays a role in this process is important since it suggests that therapeutic strategies that focus on secretase inhibition should control for the presence of the *ApoE4* allele.

Excitotoxicity is involved both in acute cell death after CNS injuries and in the neuronal death that is part of AD. Human *ApoE4* carriers have poorer recovery after head trauma or stroke [52,53] with severe neurodegeneration [54]. Some studies of transgenic mice that overexpress ApoE3 or ApoE4 suggested a protective role of ApoE3 against excitotoxicity compared to ApoE4 [55,56] whereas other studies revealed no differences [57,58]. The controversy could be due to cellular source of ApoE. Buttinit et al. showed that neuron-specific expression of ApoE4 promotes excitotoxic cortical neuron death, whereas ApoE4 expressed by astrocytes was as neuroprotective as ApoE3 [59,60]. Our findings that AD-E3/E4 BFCNs are more susceptible to glutamate induced excitotoxic death are consistent with the suggestion that neuronal ApoE4 sensitizes neurons to excitotoxicity.

The increased vulnerability of AD-E3/E4 BFCNs to glutamate-mediated cell death correlated with increased intracellular free calcium upon glutamate exposure. Recombinant truncated or full-length ApoE protein elicited rapid elevation of intracellular calcium and induced rodent cortical or hippocampal neuron death [61,62]. ApoE dose-dependently increased cytosolic free calcium concentration in the order of E4 > E3 > E2 [63]. Disruption of calcium homeostasis mediated the neurotoxic effect of ApoE4 in transgenic mice [64]. Glutamate induced calcium elevation could result from calcium influx through NMDA receptors, metabotropic glutamate receptors, activation of voltage-sensitive calcium channels (VSCCs), or mobilization of calcium from intracellular calcium stores [65]. We found calcium influx through VSCCs did not differ significantly between one control and *ApoE3/E4* line, consistent with previous findings of a lack of contribution by VSCCs to calcium mediated excitotoxicity [66,67]. Alternatively, abnormalities in the cellular calcium buffering system could also result in sustained intracellular calcium elevations.

## Conclusions

In this study we modeled sporadic Alzheimer’s disease with an *ApoE3/E4* genotype using human iPSCs that we established from age-matched control and AD-E3/E4 fibroblasts. We then differentiated the iPSCs into BFCNs to enable us to study the disease process in an identified, disease relevant population of human neurons. Using these iPSC-derived BFCNs, we found that AD-E3/E4 neurons had increased Aβ42/Aβ40 ratios, a typical biochemical feature of AD, similar to familial *presenilin* mutation lines [14,16]. However we found that lower doses of γ-secretase inhibitors surprisingly increased Aβ secretion by AD-E3/E4 BFCNs whereas they reduced Aβ secretion by control and familial AD neurons as expected. AD-E3/E4 BFCNs also exhibited increased vulnerability to normally non-lethal concentrations of calcium ionophore or L-glutamate. The excitotoxicity caused by glutamate exposure correlated with increased cytosolic free calcium in the AD-E3/E4 BFCNs upon stimulation. In summary, iPSCs-derived BFCNs from patients with AD and an *ApoE3/E4* genotype display biochemical features of the disease including an increased ratio of Aβ42/40 and increased susceptibility to cell death in response to calcium influx or exposure to glutamate. The ability to generate BFCNs with an AD phenotype is a significant step both for understanding disease mechanisms and for facilitating high throughput screening for agents that promote BFCNs survival.

## Methods

### Reprogramming and cell culture

Human age-matched control and Alzheimer’s disease fibroblasts were purchased from the Coriell Cell Repositories and ATCC. Fibroblast cultures were maintained in DMEM, 15% FBS (Fetal Bovine Serum). Fibroblasts were infected with Oct4-Klf4-Sox2-cMyc retrovirus for two rounds and split onto 75,000 cells/ml gamma-irradiated feeder cells on day 6. Medium was switched to hESC (human Embryonic Stem Cell) medium with DMEM/F12, 20% KSR (Knock-out Serum Replacement), 0.1 mM non-essential amino acids, 2 mM L-glutamine, 100 μM β-mercaptoethanol, 10 ng/ml bFGF. Medium was changed every other day until colonies were picked up around day 20 and then changed daily.

### Teratoma assay and karyotyping

All animal procedures were performed in accordance with the Public Service Policy on Human Care and Use of Laboratory Animals and all procedures were approved by the Northwestern University Institutional Animal Care and Use Committee. iPSCs were harvested as aggregates in hESC medium. Cells were injected subcutaneously through an 18-gauze needle into hind limbs of NOD-SCID mice. After 6–8 weeks, mice were euthanized and teratomas were excised, sectioned and analyzed by H&E staining. iPSCs were plated in T-25 cell culture flasks and karyotyped by Children’s Hospital, Oakland using standard G banding.

### Differentiation of iPSCs

The protocol for generating BFCNs from iPSCs was adapted from our previously described protocol for hESCs [17]. Briefly, iPSCs were split onto matrigel plates in feeder cell conditioned medium supplemented with 10 ng/ml bFGF and 10 μM RA. After 7 days, cells were harvested in hESC medium without bFGF to allow neurosphere formation. Individual neurospheres were then picked into neurosphere medium (DMEM/F12, 0.1 mM non-essential amino acids, 1× N2 supplement, 100 μM β-mercaptoethanol, 8 μg/ml heparin, 20 ng/ml bFGF, 20 ng/ml EGF) for another 4 days. 100 ng/ml FGF8 and 200 ng/ml SHH were added for 72 hours at which point neurospheres were dissociated and nucleofected with 4 μg Lhx8/Gbx1-IRES-GFP overexpression plasmid (for control, GFP plasmid was used). 48–72 hours later, cells were fac-sorted for GFP and plated in neurobasal medium with 1× B-27 supplement, 2 mM glutamax, 2 mM glutamine, 20 ng/ml bFGF, 100 ng/ml NGF. Cytosine arabinoside was added from day 5 to day 10 at a concentration of 2.4 μM to eliminate proliferation of contaminating non-neuronal cells.

### Retrovirus production

The four human reprogramming factors were cloned into a single retroviral vector linked by 2A sequences: pMXs-Oct4-p2A-Klf4-t2A-Sox2-e2A-cMyc (pMXs-OKSM). 293T cells were co-transfected with pMXs-OKSM, pCMV-VSVG and pCMV-gag/pol. Supernatants from day 2 and 3 were used to directly infect human fibroblasts in the presence of 5 ng/ml polybrene.

### Quantitative PCR (Q-PCR)

RNA was extracted with an RNAequeous-Micro kit (Ambion). A total of 500 ng RNA was used for cDNA synthesis with the Thermoscript system (Invitrogen). 1 μl of cDNA was mixed with SYBR green master mix (Applied Biosystems) and the PCR reaction was carried out in a Realplex^2^ Mastercycler (Eppendorf) using the following cycling parameters: 95°C 15 s, 60°C 60 s for 40 cycles. Melting curves were generated to confirm the presence of one single product.

### ELISA and western blot

BFCNs were conditioned in neuron medium (see above) for 72 hours. Human Aβ40 and Aβ42 ELISA (Invitrogen) were carried out simultaneously according to the manufacturer’s instructions with the conditioned medium. Cellular protein was harvested with M-PER protein extraction reagent with 1:500 Halt protease inhibitor cocktail (Thermo Scientific). Protein concentration was determined by BCA assay. For western blot analyses, protein samples were separated by SDS-PAGE on 4-20% Tris-glycine gradient gels (Invitrogen), and then transferred onto PDF (polyvinylidene difluoride) membranes (Millipore). Membranes were blocked with 5% milk and then incubated with primary antibodies overnight at 4°C. HRP (Horseradish peroxidase)-conjugated secondary antibodies and ECl substrate (Thermo Scientific) were used for detection. Primary antibodies included: Anti-Cav1.2 (1:200, Neuromab).

### Neurotoxicity assays

Neurons were treated with DMSO, 250 nM, 1 μM or 15 μM ionomycin for 16 hours and Live/Dead Viability/Cytotoxicity assays (Invitrogen) were performed. Cells were incubated in 2 μM calcein and 4 μM ethidium homodimer-1 for 30 minutes at room temperature and then immediately mounted and imaged using Zeiss microscopy. For the glutamate excitotoxicity assay, 50 μM glutamate or saline dissolved in 2 mM calcium buffer was added to 2-week neuron cultures for 15 minutes and washed twice with PBS before adding culture medium back in for another 24 hours. MTT assay from Promega was performed to determine cell viability according to manufactures’ instructions.

### Immunocytochemistry

Cells were fixed in 4% PFA for 15 minutes followed by two 1xPBS washes. Cells were blocked by 10% bovine serum in 1xPBS at room temperature for 1 hour. Primary antibodies were applied for overnight at 4°C in 1xPBS containing 1% BSA and 0.25% Triton-X-100. Following three washes, alexa fluor conjugated secondary antibodies (1:1000, invitrogen) together with DAPI (1:2000) were added for 1 hour. After three more washes, coverslips were mounted with Prolong Gold antifade reagent (Invitrogen) and imaged. Primaries antibodies used were: Oct4 (1:1000, Santa Cruz), Sox2 (1:1000, Millipore), Ssea4 (1:500, Chemicon), Tra1-60 (1:500, Chemicon), Collagen type IV (1:500, Abcam), Gata4 (1:1000, Santa Cruz), Map2 (1:1000, Abcam), Gli1 (1:500, Santa Cruz), Forse1 (1:300, DSHB), Nkx2.1 (1:500, Abcam), Pax6 (1:1000, DHSB), Mash1 (1:1000, BD Pharmingen), p75-NTR (1:1000, Abcam), VAChT (1:1000, SYSY), HB9 (1:1000, Abcam) and ChAT (1:600, Aves Labs).

### Calcium imaging

2-week old neurons grown on PDL-coated glass coverslips were incubated with 2 μM calcium-sensitive dye Fura-2 (Invitrogen) in calcium buffer for 30mins and washed three times afterwards. Neurons were incubated for an additional 30 mins before imaging. Coverslips were mounted onto a chamber and placed on the stage of a laser confocal microscope. A 20× objective was used and the imaging was done at room temperature. Signals were collected at 3 s intervals from wavelengths 340 nm and 380 nm and the ratio of 340 nm/380 nm was recorded. Recordings were allowed to go for 10–15 minutes to establish baseline. 56 mM KCl or 50 μM glutamate were added for 1 min to elicit calcium influx. For measurement of cytosolic free cacium, 10 μM ionomycin was added to zero calcium buffer to deplete intracellular calcium stores and to 20 mM calcium buffer to get a maximal calcium level. Imaging buffers were adjusted to PH value of 7.2-7.4 and osmolality of 300–310. Detailed description of buffers are listed below: Calcium buffer: 125 mM NaCl, 3 mM KCl, 2 mM CaCl2, 25 mM glucose, 10 mM Hepes; Zero calcium buffer: 125 mM NaCl, 3 mM KCl, 25 mM glucose, 10 mM Hepes, 10 mM EDTA; High calcium buffer: 100 mM NaCl, 3 mM KCl, 25 mM glucose, 10 mM Hepes, 20 mM CaCl2.

### Electrophysiology

Cultured neurons were incubated for 15 min at 37°C in oxygenated standard artificial CSF [ACSF] containing [in mM]: 130 NaCl, 24 NaHCO3, 3.5 KCl, 1.25 NaH2PO4, 1.5 CaCl2, 1 MgSO4, and 10 glucose, saturated with 95% O2 and 5% CO2 at pH 7.4 and then transferred to the recording chamber in oxygenated standard ACSF. Whole cell patch clamp recordings were performed and observed with the aid of microscope [BX-50WI; Olympus] and visualized with a chilled charge-coupled device video camera [Dage-MTI] with a 40× water-immersion differential interference contrast objective. For whole cell voltage clamp recordings, patch electrodes with a resistance of 5–7 M were pulled from borosilicate capillaries [World Precision Instruments; PG52165-glass]. Patch pipettes were filled with a solution of [in mM] 150 KCl, 10 HEPES, 4 Mg2ATP, 0.5 NaGTP, and 10 phosphocreatine. The pH was adjusted to 7.3 with KOH. Whole-cell voltage clamped recordings were obtained from neurons using an Axopatch 200B patch-clamp amplifier [Molecular Devices] and the data were captured with pClamp 9.0 software [Molecular Devices]. Voltage-dependent Na currents was recorded under voltage clamp conditions and blocked by bath application of Tetrodotoxin (TTX; 2 μM, Tocris Bioscience; R&D Systems, Inc. MN). Data analysis. Data were filtered at 2 kHz and digitized at 10 kHz using a Digidata 1322A analog-to-digital board. Analysis was performed using the pClamp 9.0 [Molecular Devices].

### Statistics

GraphPad software was used for statistical analyses. Student’s t-test or ANOVA with the Bonferroni post test was used to generate P values.

## Abbreviations

iPSCs: Induced Pluripotent Stem Cells; hESCs: Human Embryonic Stem Cells; BFCNs: Basal Forebrain Cholinergic Neurons; VSCC: Voltage-sensitve Calcium Channels; SHH: Sonic Hedgehog; FGF8: Fibroblast Growth Factor 8; MGE: Medial Ganglion Eminence; NGF: Nerve Growth Factor; ChAT: Choline Acetyltransferase; VAChT: Vesicular Acetylcholine Transferase; ApoE: Apolipoprotein E.

## Competing interests

The authors declare that they have no competing interests.

## Authors’ contributions

LD: conception and design, collection and assembly of data, data analysis and interpretation, manuscript writing; BB: collection and assembly of data, data analysis and interpretation; AB: collection and assembly of data, data analysis and interpretation; LP: collection and assembly of data; RM: conception and design, data analysis and interpretation, final approval of manuscript; JK: conception and design, financial support, administrative support, data analysis and interpretation, manuscript writing, final approval of manuscript.

## Supplementary Material

Additional file 1: Figure S1 Polycistronic reprogramming vector map and primer sequences. **(A)** Reprogramming factors in the order of Oct4, Klf4, Sox2 and c-Myc were cloned into the MMLV-based retroviral backbone pMXs linked by viral 2A sequences, **(B)** Oct4 and Klf4, Sox2 and c-Myc were cloned using recombinant PCR and the resulting two fragments were ligated via XbaI restriction site. Primers used are listed as shown. Click here for file

Additional file 2: Figure S2 Induced pluripotent stem cell characterization. **(A)** Undifferentiated iPSC colonies stained positive for the pluripotent stem cell markers, Oct4, Sox2 (red), Ssea4 and Tra1-60 (green). Nuclear staining with DAPI is in blue. Scale bar = 85 μm. (control 2 line shown). **(B)** Following embryonic body differentiation, iPSCs gave rise to cell types positive for Map2 (green, ectoderm), Gata4 (red, endoderm) and Collagen type IV (green, mesoderm). Scale bar = 85 μm. (AD-E3/E4 line 4402 shown). **(C)** iPSCs formed teratomas when injected into mice. H&E staining revealed characteristic morphologies of all three germ layers. Scale bar = 313 μm. (AD-E3/E4 line 11414 shown). Click here for file

Additional file 3: Figure S3 Flow cytometry sorting for Lhx8/Gbx1/EGFP plasmid nucleofected neural progenitor cells. Representative dot plots of flow cytometry sorting for Lhx8/Gbx1/EGFP nucleofected neural progenitor cells. **(A)** Dots (cells) within region R1 were live cells, this portion of cells were further sorted for GFP. **(B)** Dots within region R3 were GFP positive cells, indicating cells had successfully taken up the plasmid with Lhx8 and Gbx1 genes in it. GFP positive cells were sorted and plated for further experiments. Click here for file

Additional file 4: Figure S4 Basal forebrain cholinergic neuron generation from AD iPSCs. **(A)** AD-E3/E4 line 11414 neurosphere sections after SHH and FGF8 treatment stained positive for forebrain marker FoxG1 (green) and ventral marker Nkx2.1 (red). **(B)** Representative confocal image of neurons generated from AD-E3/E4 line 11414 stained with neuronal marker Map2 (red) and basal forebrain cholinergic marker p75-NTR (green). Nuclear staining with DAPI is in blue. Scale bar = 85 μm. **(C)** Quantification and comparison of generation efficiency of ventral forebrain precursors, represented by the ratios of Nkx2.1, Mash1 and FoxG1 positive cells to total DAPI positive cells respectively. **(D)** Quantification and comparison of neuron generation efficiency, represented by the ratios of Map2 positive cells to total DAPI positive cells; and final basal forebrain cholinergic neuron purity counted as the ratio of ChAT positive neurons to Map2 positive total neurons. There are no statistically significant differences among control and AD lines in the ability of generating ventral forebrain precursors or basal forebrain cholinergic neurons (n = 3, mean ± SEM, two-way ANOVA, p = 0.9665 and p = 0.4701 respectively). Click here for file

Additional file 5: Figure S5 Control and AD BFCNs express L-type voltage gated calcium channel. Western blot analysis for expression of voltage gated calcium channel by BFCNs in lines control1, 11414 and 4402-1. An anti-Cav1.2 monoclonal antibody was used to detect the α subunit of voltage gated calcium channel at around 240 kD (upper panel). GAPDH was also probed for loading control (lower panel). Click here for file

Additional file 6: Figure S6 Live/dead assay for ionomycin induced neuronal toxicity. 2-week old BFCNs from either AD **(A)** or control **(B)** lines were treated with DMSO, 1 μM or 15 μM ionomycin for 16 hours, live/dead assay was performed. Live cells were stained with green and counted. Click here for file
