# Supplementary figures and images for: Stem cell derived basal forebrain cholinergic neurons from Alzheimer’s disease patients are more susceptible to cell death
Source: Mol Neurodegener. 2014 Jan 8;9:3. doi: 10.1186/1750-1326-9-3 (PMC3896712; doi:10.1186/1750-1326-9-3)

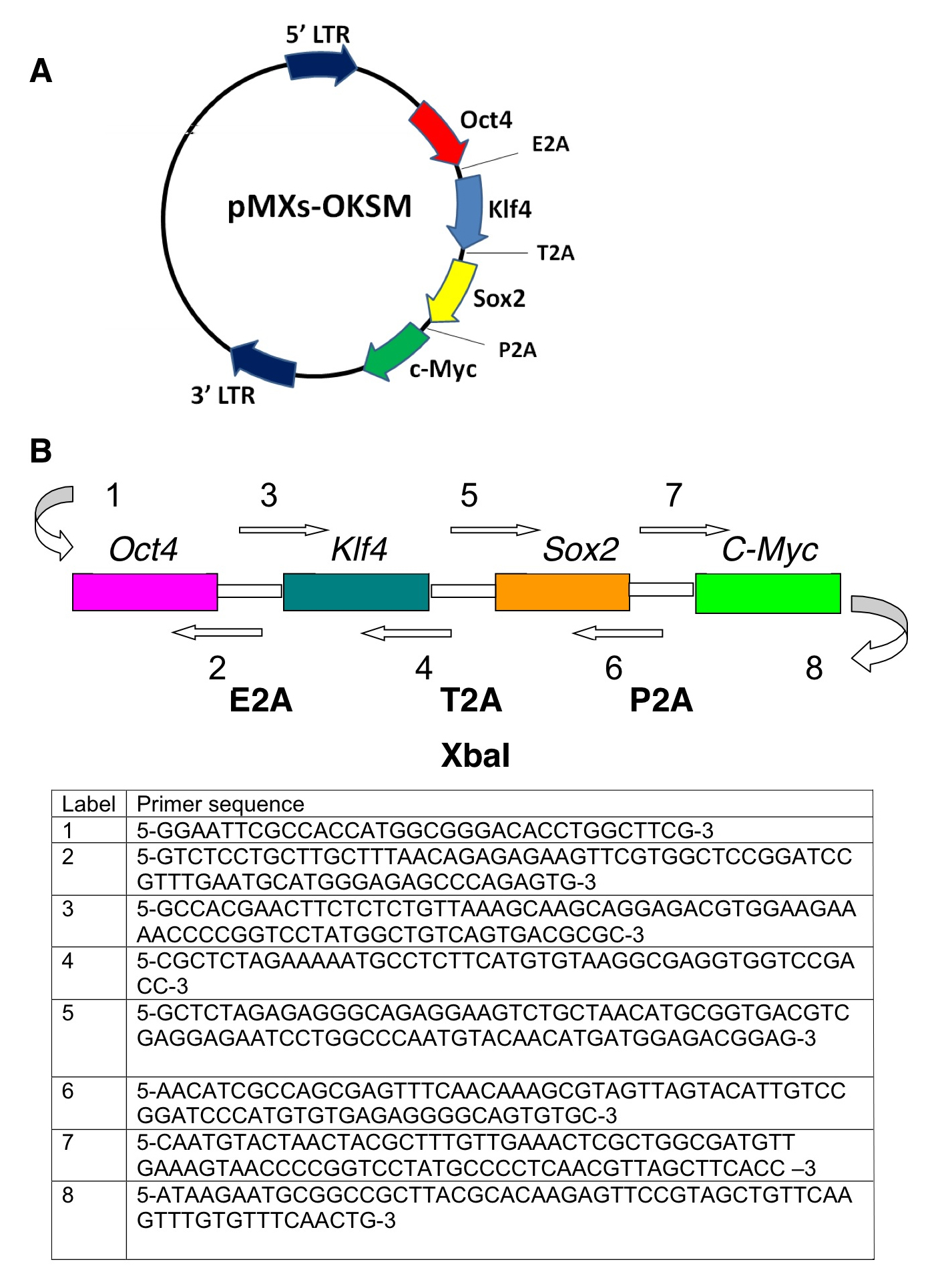

Supplement: Additional file 1: Figure S1 — Polycistronic reprogramming vector map and primer sequences. (A) Reprogramming factors in the order of Oct4, Klf4, Sox2 and c-Myc were cloned into the MMLV-based retroviral backbone pMXs linked by viral 2A sequences, (B) Oct4 and Klf4, Sox2 and c-Myc were cloned using recombinant PCR and the resulting two fragments were ligated via XbaI restriction site. Primers used are listed as shown. [file 1750-1326-9-3-S1.jpeg]

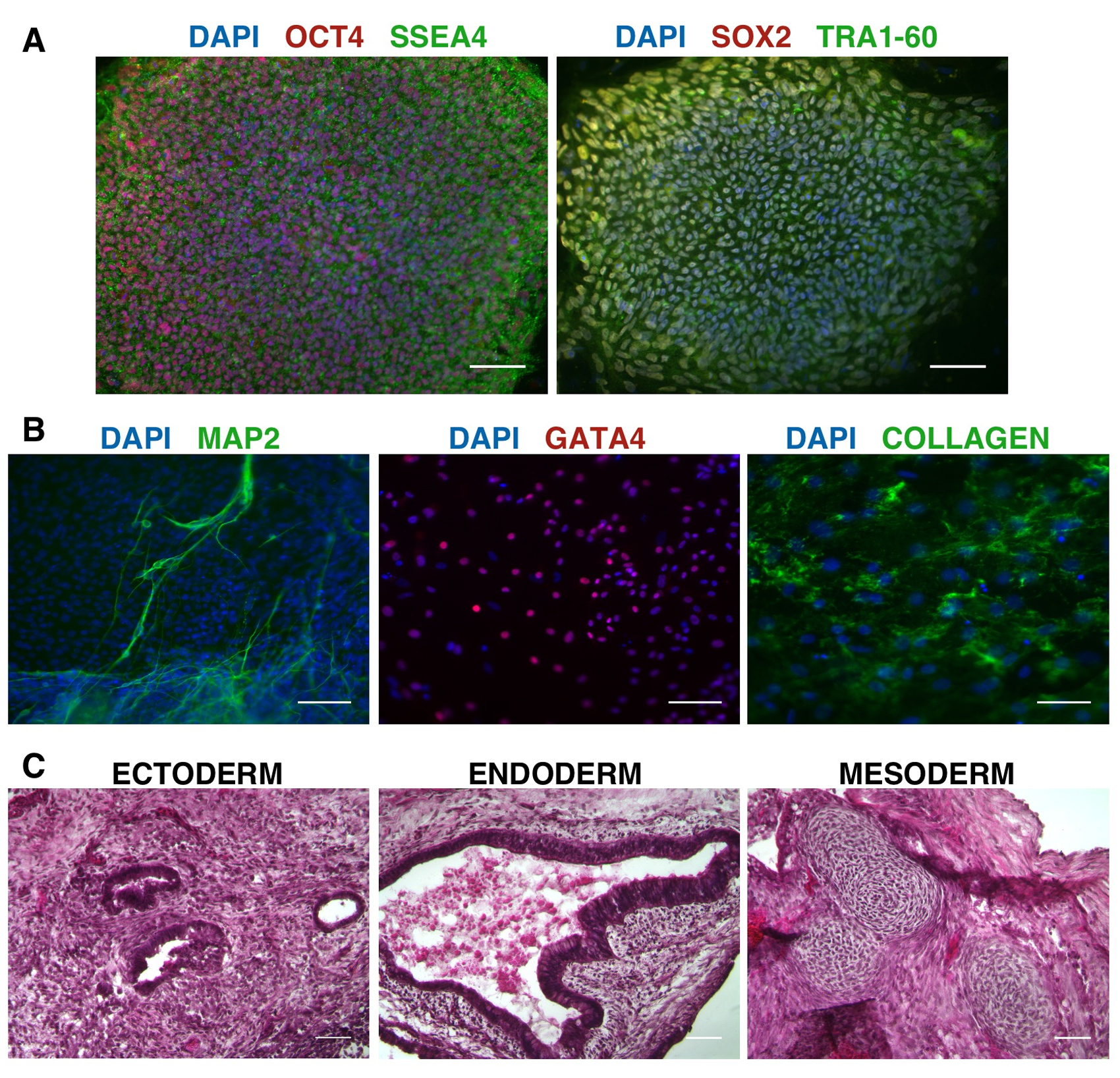

Supplement: Additional file 2: Figure S2 — Induced pluripotent stem cell characterization. (A) Undifferentiated iPSC colonies stained positive for the pluripotent stem cell markers, Oct4, Sox2 (red), Ssea4 and Tra1-60 (green). Nuclear staining with DAPI is in blue. Scale bar = 85 μm. (control 2 line shown). (B) Following embryonic body differentiation, iPSCs gave rise to cell types positive for Map2 (green, ectoderm), Gata4 (red, endoderm) and Collagen type IV (green, mesoderm). Scale bar = 85 μm. (AD-E3/E4 line 4402 shown). (C) iPSCs formed teratomas when injected into mice. H&E staining revealed characteristic morphologies of all three germ layers. Scale bar = 313 μm. (AD-E3/E4 line 11414 shown). [file 1750-1326-9-3-S2.jpeg]

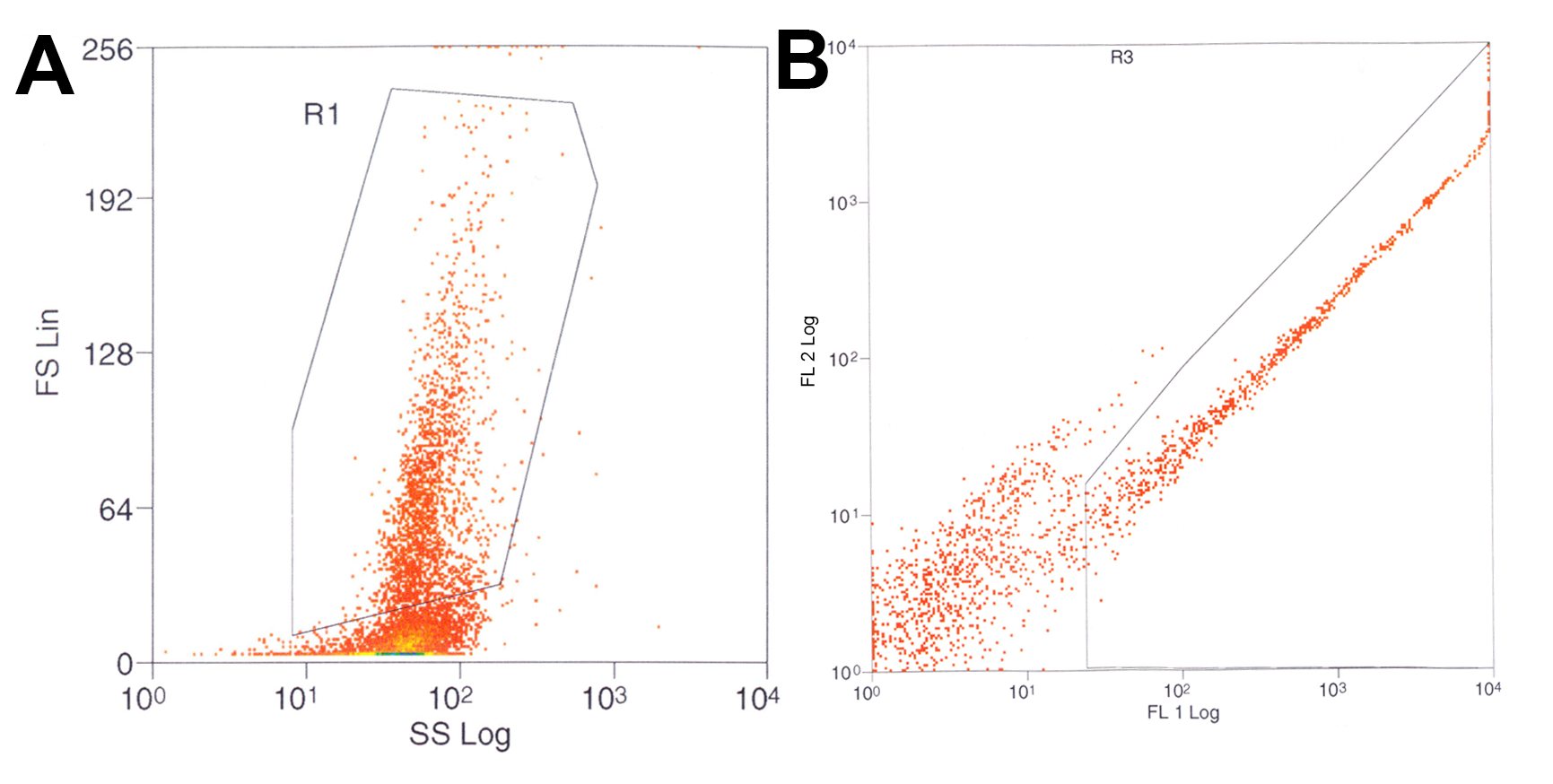

Supplement: Additional file 3: Figure S3 — Flow cytometry sorting for Lhx8/Gbx1/EGFP plasmid nucleofected neural progenitor cells. Representative dot plots of flow cytometry sorting for Lhx8/Gbx1/EGFP nucleofected neural progenitor cells. (A) Dots (cells) within region R1 were live cells, this portion of cells were further sorted for GFP. (B) Dots within region R3 were GFP positive cells, indicating cells had successfully taken up the plasmid with Lhx8 and Gbx1 genes in it. GFP positive cells were sorted and plated for further experiments. [file 1750-1326-9-3-S3.tiff]

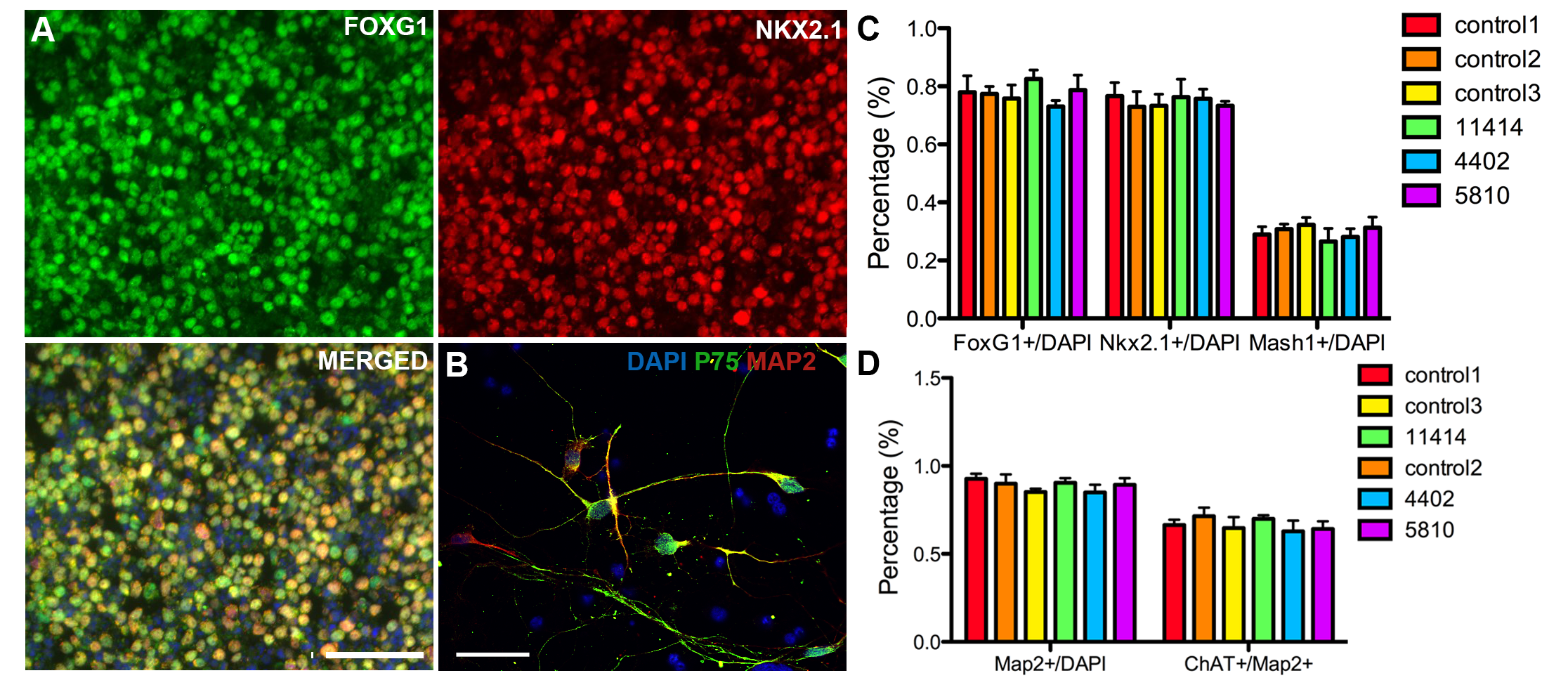

Supplement: Additional file 4: Figure S4 — Basal forebrain cholinergic neuron generation from AD iPSCs. (A) AD-E3/E4 line 11414 neurosphere sections after SHH and FGF8 treatment stained positive for forebrain marker FoxG1 (green) and ventral marker Nkx2.1 (red). (B) Representative confocal image of neurons generated from AD-E3/E4 line 11414 stained with neuronal marker Map2 (red) and basal forebrain cholinergic marker p75-NTR (green). Nuclear staining with DAPI is in blue. Scale bar = 85 μm. (C) Quantification and comparison of generation efficiency of ventral forebrain precursors, represented by the ratios of Nkx2.1, Mash1 and FoxG1 positive cells to total DAPI positive cells respectively. (D) Quantification and comparison of neuron generation efficiency, represented by the ratios of Map2 positive cells to total DAPI positive cells; and final basal forebrain cholinergic neuron purity counted as the ratio of ChAT positive neurons to Map2 positive total neurons. There are no statistically significant differences among control and AD lines in the ability of generating ventral forebrain precursors or basal forebrain cholinergic neurons (n = 3, mean ± SEM, two-way ANOVA, p = 0.9665 and p = 0.4701 respectively). [file 1750-1326-9-3-S4.tiff]

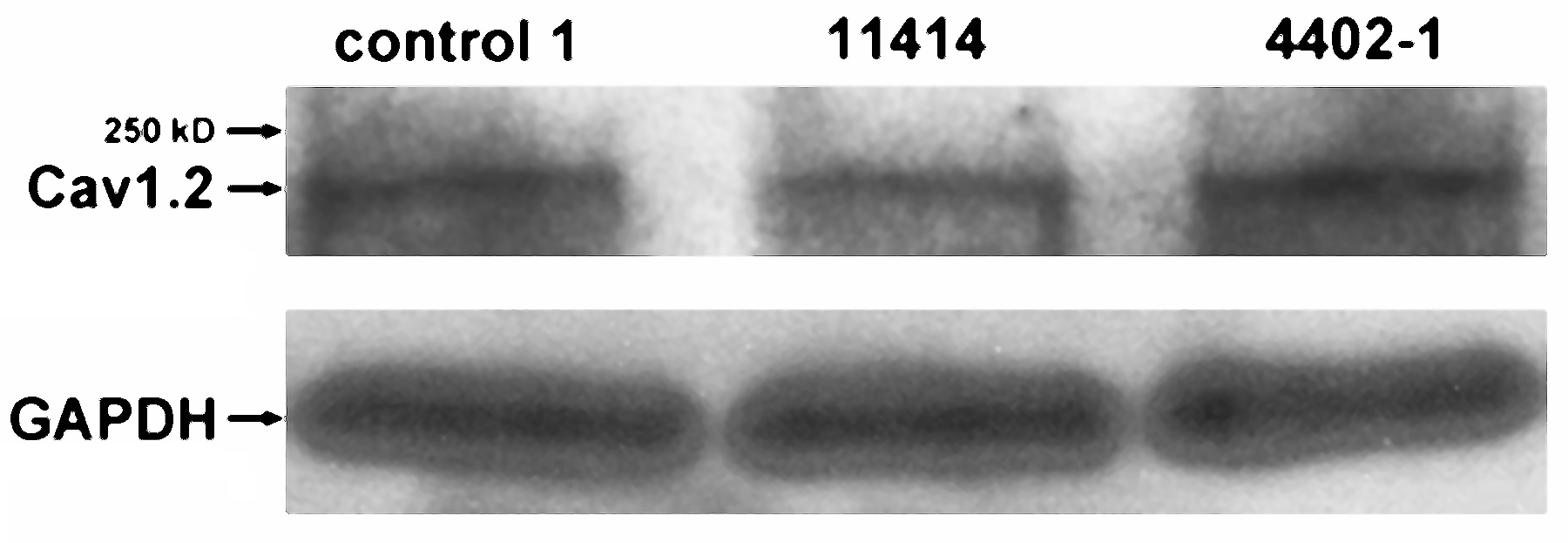

Supplement: Additional file 5: Figure S5 — Control and AD BFCNs express L-type voltage gated calcium channel. Western blot analysis for expression of voltage gated calcium channel by BFCNs in lines control1, 11414 and 4402-1. An anti-Cav1.2 monoclonal antibody was used to detect the α subunit of voltage gated calcium channel at around 240 kD (upper panel). GAPDH was also probed for loading control (lower panel). [file 1750-1326-9-3-S5.tiff]

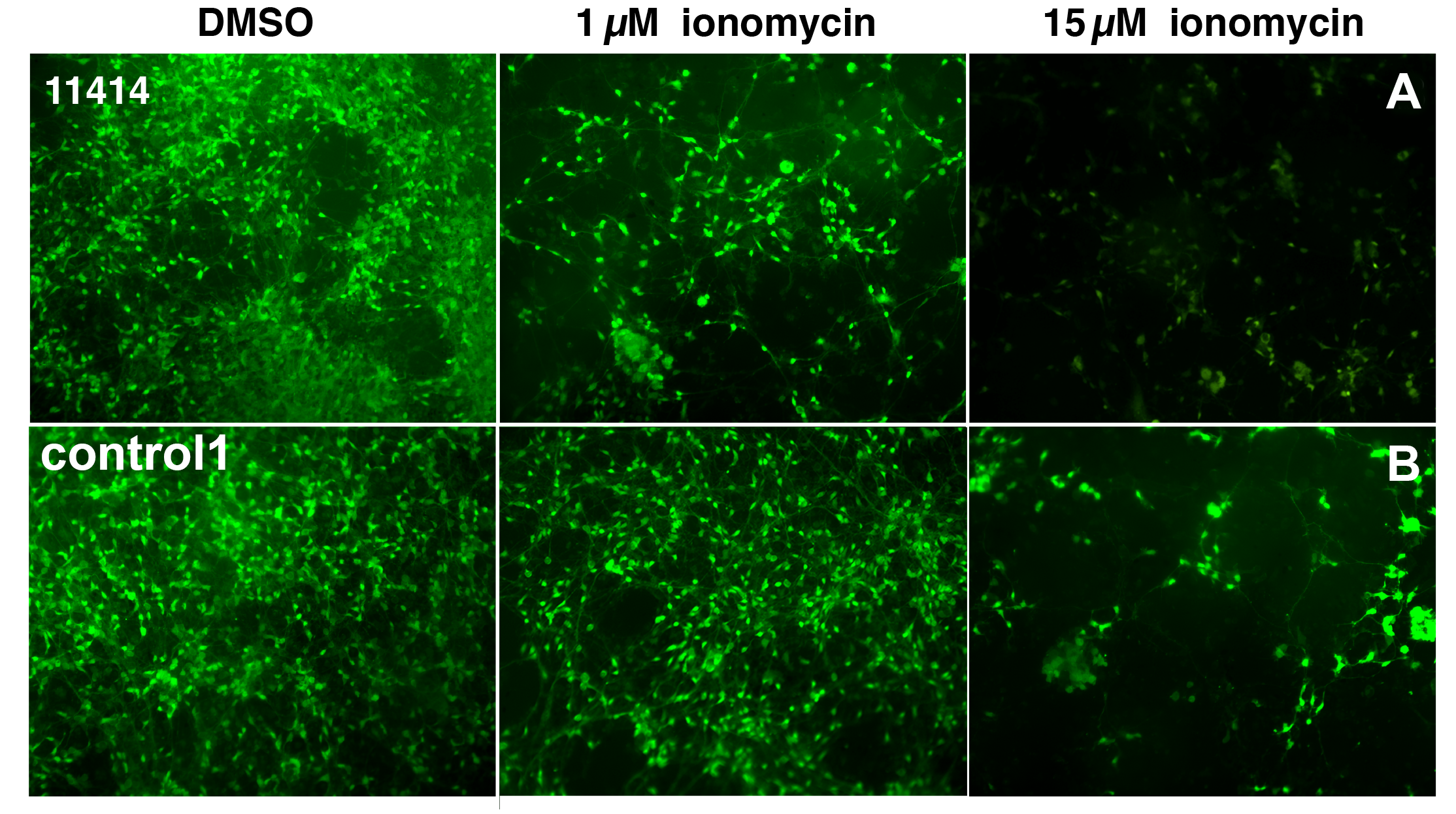

Supplement: Additional file 6: Figure S6 — Live/dead assay for ionomycin induced neuronal toxicity. 2-week old BFCNs from either AD (A) or control (B) lines were treated with DMSO, 1 μM or 15 μM ionomycin for 16 hours, live/dead assay was performed. Live cells were stained with green and counted. [file 1750-1326-9-3-S6.tiff]
